# T Cell Targeting by Anthrax Toxins: Two Faces of the Same Coin

**DOI:** 10.3390/toxins3060660

**Published:** 2011-06-20

**Authors:** Silvia Rossi Paccani, Cosima T. Baldari

**Affiliations:** 1 Department of Evolutionary Biology, University of Siena, Via Aldo Moro 2, 53100 Siena, Italy; Email: baldari@unisi.it; 2 Novartis Vaccines, Via Fiorentina 1, 53100 Siena, Italy

**Keywords:** lethal toxin, edema toxin, T cell, Th subset, TCR signaling, cAMP, MAP kinases, immunosuppression, immunodeviating

## Abstract

*Bacillus anthracis*, similar to other bacterial pathogens, has evolved effective immune evasion strategies to prolong its survival in the host, thus ensuring the unchecked spread of the infection. This function is subserved by lethal (LT) and edema (ET) toxins, two exotoxins produced by vegetative anthrax bacilli following germination of the spores. The structure of these toxins and the mechanism of cell intoxication are topics covered by other reviews in this issue. Here we shall discuss how *B. anthracis* uses LT and ET to suppress the immune defenses of the host, focusing on T lymphocytes, the key players in adaptive immunity. We shall also summarize recent findings showing that, depending on its concentration, ET has the ability not only to suppress T cell activation but also to promote the polarization of CD4^+^ T cells to the Th2 and Th17 subsets, highlighting the potential use of this toxin as an immunomodulator.

## 1. Introduction

Bacteria have developed a variety of strategies to hamper or modify the host immune response to establish a productive infection. *Bacillus anthracis *has a challenging interaction with the host in that it exploits macrophages, the first line of defense of the innate immunity, to reach the heart of the immune system, the lymph node. *B. anthracis* invades the host through one of three known portals—upper airways, gastrointestinal tract or skin—in the form of metabolically inert spores which must germinate to become pathogenic bacilli. This crucial function is subserved by phagocytes patrolling the tissues, such as alveolar macrophages or lung dendritic cells (DC) in inhalational anthrax. Following their ingestion by the phagocytes, the spores are carried through the lymphatics to the regional lymph nodes, where they germinate to become vegetative bacilli that are first released locally and eventually enter the bloodstream [1]. 

Pathogenic strains of *B. anthracis* produce a deadly tripartite toxin, which is composed of protective antigen (PA), lethal factor (LF) and edema factor (EF) [2]. The three toxin components combine to form two binary toxins, the lethal toxin (LF+PA) and the edema toxin (EF+PA), that help the bacterium to evade the immune system and eventually kill the host if the infection becomes systemic. PA consists of four domains, of which the *C*-terminal domain is released upon proteolytic cleavage by a furin-like membrane protease or by a serum protease, resulting in oligomerization of truncated PA into heptamers or octamers that bind EF and LF. The PA-EF or the PA-LF complexes are then endocytosed inside acid compartments. The low pH of the endosomal lumen induces a conformational change of the complex which results in the insertion of PA into the lipid bilayer and translocation of LF and EF to the cytosol [3]. LF is a zinc metalloprotease which cleaves and inactivates several members of the MAPKK family [4,5,6]. EF is a Ca^2+^/calmodulin dependent adenylate cyclase which produces large amounts of the second messenger cAMP [7,8]. By interfering with the signaling pathways activated by a variety of surface receptors the anthrax toxins dismantle the first line of defense of the immune system [1,9]. LT and ET blunt in fact the production and release of proinflammatory mediators by macrophages and promote their apoptosis, thus preventing polymorphonuclear cell recruitment to the site of infection and pathogen clearance [1,9,10,11,12]. Furthermore both toxins impair DC activation and maturation, thus preventing the initiation of adaptative immunity [13]. 

While the impairment of DC functions could efficiently prevent *per se* mounting of the adaptive immune response, *B. anthracis* also directly targets the central regulators of adaptive immunity, *i.e.*, B and T lymphocytes. LT dramatically reduces B cell proliferation and impairs immunoglobulin production [14]. Moreover by targeting T lymphocytes, which are the specific subject of this review, *B. anthracis* ensures a complete coverage of all the actors responsible for the immune defenses of the host. 

## 2. T Cells as Targets of the Anthrax Toxins

Although spore germination, and hence toxin production, is believed to occur in the regional lymph nodes, a recent report has challenged this view. Using *in vivo* imaging of infected mice, Glomski *et al.* have provided evidence that the spores germinate and establish initial infections at the site of inoculation in both inhalational and gastrointestinal infections, in nasal-associated lymphoid tissues and Peyer’s patches, respectively, without needing to be transported to the draining lymph nodes [15]. While the concentrations of LT and ET have been measured to date only in the blood during systemic infection [16], this important finding suggests that the anthrax toxins can accumulate locally to levels sufficiently high to interfere with the function not only of the resident phagocytes, but also of the immune cells present in the mucosa associated lymphoid tissue, including T cells. 

The identification of the anthrax toxin receptors (ANTXR1 and ANTXR2) [17,18,19] provided support to the notion that not only are T cells suitably located to be targeted by the toxins, but also have the means to become effectively intoxicated. T cells express indeed both TEM8 (ANTXR1) and CMG2 (ANTXR2), the latter being the prevalent form, at least in mouse splenic CD4^+^ and CD8^+^ T cells [18,20]. After their delivery to the cytosol, the catalytic subunits of LT and ET have the potential to subvert the signaling pathways that control the activation of naïve T cells and their subsequent differentiation to armed effectors by interfering with the MAPK and cAMP/PKA dependent signaling cascades, which are centrally implicated in these processes [21,22]. 

## 3.LT and ET Suppress T Cell Activation

Both LT and ET potently suppress human T lymphocyte activation *in vitro*, as assessed by surface expression of activation markers (CD69, CD25), cytokine secretion (IL-2, TNF-α, IFN-γ, IL-5) and proliferation [23,24]. This direct inhibitory activity on CD4^+^ T cells has been also reported in the mouse, where the toxins have been shown to impair T cell antigen receptor (TCR) mediated T cell activation, cytokine production (IL-3, IL-4, IL-5, IL-6, IL-10, IL-17, GM-CSF, TNF-α, IFN-γ) and proliferation both *in vitro* and *ex vivo* after administration of sublethal doses of LT or ET [25]. A similar effect has recently been observed on CD1d-restricted NKT cells, which play an important role in the orchestration of a protective adaptive response against pathogens [26]. Of note, LT and ET synergize in suppressing T cell activation at concentrations which alone elicit minor effects [23]. This is likely to be of relevance at the onset of infection, when the local toxin concentrations may be too low to effectively target the immune cells. It should be emphasized that immunological memory in survivors of bioterrorism related inhalational anthrax (measured as anti-PA antibody titers) [27], as well as CD4^+^ T cell responses in individuals previously naturally infected with cutaneous anthrax [28], are functional several months or years after successful antibiotic treatment of the infection. This indicates that the impairment in adaptive immunity observed following T cell treatment with LT or ET *in vitro*, or short-term *in vivo* administration of the toxins to mice, is not long-lasting and may be instrumental only at the onset of infection to ensure survival of the vegetative bacilli until colonization. 

**Figure 1:**
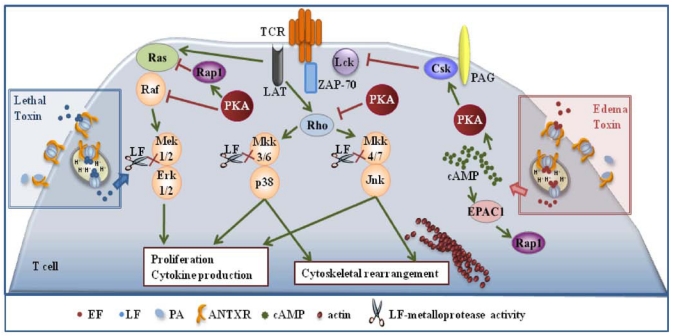
Effects of the anthrax toxins on T cell activation. Lethal (LT) and edema (ET) block T cell activation and proliferation by interfering with T cell antigen receptor (TCR) signaling. LT does so directly by cleaving MAPKKs, while the effect of ET is mediated by molecular targets of its cAMP elevating activity.

Inihibition of T cell activation by LT and ET is dependent on the respective enzymatic activities. Commitment to T cell activation is characterized by the temporally regulated expression of a subset of genes, which eventually leads to the proliferation of antigen specific T cells and acquisition of effector functions. Both the classical MAPK cascade, whose endpoint is Erk [22], and the stress kinase cascades, whose endpoints are p38 and JNK [29], are implicated in coupling the TCR to this complex gene expression cascade. LT and ET have both the potential to disable these pathways. LT can do so directly by cleaving MAPKKs, while the effect of ET is mediated by molecular targets of its cAMP elevating activity. Among these, PKA has a prominent role. By producing cAMP, ET activates PKA, which impairs Ras activation indirectly by activating the Ras antagonist, Rap1, and moreover inactivates the first MAPK in the cascade, Raf, by phosphorylating an inhibitory serine residue. PKA impairs moreover the activation of the stress kinases by blocking the activation of the small GTPase Rho [30] (Figure 1). Hence MAPKs represent a point of convergence of the intracellular activity of the two anthrax toxins. Interestingly, after release to the cytosol from late ensodomes, ET remains bound to the these organelles [31], thereby generating high levels of cAMP compartmentalized close to this location, which results in the induction of waves of activated PKA emanating from perinuclear foci [32]. This suggests that not only is PKA activated to abnormally high and sustained levels in the presence of ET, but that pools of PKA which are normally not implicated in the control of TCR signaling may be mobilized under these conditions. 

It must be emphasized that ET has the potential not only to impair the MAPK cascades, but also to antagonize TCR signaling beginning from the earliest steps. Initiation of TCR signaling is crucially dependent on phosphorylation of the intracellular tails of the the CD3 complex subunits by the tyrosine kinase Lck. This results in the generation of docking sites for the second tyrosine kinase in the cascade, ZAP-70 which, when bound to CD3ζ, becomes activated and in turn phosphorylates a number of key substrates. Among these is the transmembrane adaptor LAT, which forms a scaffold for the assembly of a multimolecular signaling complex that couples the TCR to downstream events [33]. The activity of Lck is negatively regulated by the kinase Csk, which is potentiated by PKA (Figure 1). By promoting the activation of PKA through its cAMP elevating activity ET may contribute to maintaining Lck in an inactive state, thereby preventing firing of the TCR signaling cascade. This has been found to be indeed the case. ET prevented TCR mediated Lck activation and CD3ζ phosphorylation in human peripheral T cells, which resulted in a generalized defect in downstream signaling [34]. This implies that ET has not only the potential to specifically target the MAPK cascades, but can cripple TCR signaling as a whole.

## 4. LT and ET Impair T Cell Chemotaxis

Orchestration of the immune response is crucially dependent on immune cell migration and homing, which is finely tuned by chemokines [35]. Naive T lymphocytes recirculate continuously from the bloodstream to lymphoid organs. There they sample APCs which have migrated from the site of infection after uptake of the pathogen or its secreted products. T cells that have encountered an APC bearing MHC bound cognate antigen stop, become activated, proliferate and differentiate to armed effectors. Effector T cells then exit the lymphoid organs and patrol the body in search of the site where the invading pathogen is located. In response to certain chemokines, T cells migrate to the inflamed tissue and cooperate with the phagocytes to clear the infection [36]. 

**Figure 2:**
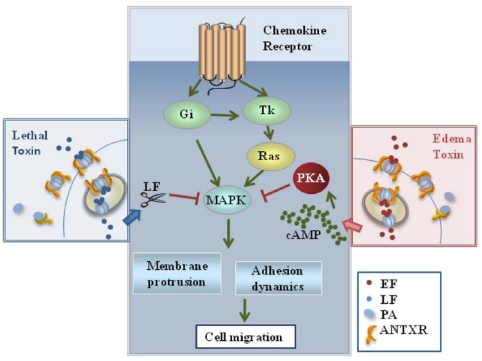
Effects of the anthrax toxins on T cell chemotaxis. LT and ET inhibit T cell chemotaxis by disrupting the MAP kinase dependent pathways orchestrated by chemokine receptors which control the dynamics of the actin cytoskeleton.

Chemotaxis is orchestrated by chemokine receptors, which are seven spanning transmembrane receptors coupled to heterotrimeric Gi proteins that modulate the activity of adenylate cyclases. These receptors activate MAPKs through their inhibitory activity on cAMP production. Furthermore, they initiate a tyrosine kinase dependent pathway leading to the activation of MAPKs [35]. Pathways initiated by chemokine receptors are therefore potential targets of the two anthrax toxins. LT and ET have indeed been found to block T cell chemotaxis [37] (Figure 2). This inhibition correlates with suppression of MAPK activation in response to the chemokines. Interestingly, both toxins also inhibit macrophage chemotaxis [37]. Hence, by inhibiting migration of both APC and T cells through disruption of MAPK signaling, *B. anthracis* can prevent the initiation of adaptive immune responses upstream of antigen presentation. Furthermore, it can suppress recruitment of phagocytes and effector cells to the site of infection. This is likely to be very relevant to cutaneous anthrax, where impaired chemotaxis might account for the observed delay in bacterial clearance and wound healing [38].

## 5. Immunodeviating Activity of ET on CD4^+^ T Cells

Dependent on the environment to which they are exposed upon encounter with antigen presented by APCs, naïve CD4^+^ T cells enter a complex differentiation program which leads to the development of at least three effector subsets with distinct and complementary properties, known as Th1, Th2 and Th17. Th1 cells are generally responsible for cell-mediated immunity, enhancing the function of macrophages by producing pro-inflammatory cytokines. Th17 cells are a recently described proinflammatory subset that complements the function of Th1 cells by promoting the recruitment and activation of phagocytes. The Th2 subset is mainly responsible for optimizing the humoral response by promoting the maturation of activated B cells to antibody-producing plasma cells [39,40,41]. While Th subsets have evolved to coordinate the adaptive response to pathogens, an alteration in their balance can result in immune related pathological outcomes. The link between Th2 cells and bronchial asthma, or the association of Th1 and Th17 with autoimmune diseases, are striking examples of the consequences of an aberrant bias towards a specific Th subset [39]. As such, Th subsets are relevant targets both in the treatment of various immune related diseases and in vaccination. 

Helper T cell subsets can be identified based on unique cytokine profiles, which are dictated by master transcription factors. These highly specific developmental programs are set during priming by the APC, which provides not only MHC bound antigen, but also developmental cues in the form of cytokines or lipid mediators. The latters include PGE_2_, which depending on the context can either suppress T cell activation or promote their differentiation and/or expansion to the Th2 subset, an activity which has recently been extended to the Th17 subset [42,43,44,45,46,47]. This results from the ability of the PGE_2_ receptors to induce cAMP accumulation and PKA activation. Although the cytokines driving the differentiation of the three subsets are known, very little is known about how the TCR signaling cascade is modulated to result in developmental outcomes so profoundly different. Nevertheless, PKA has been implicated as a negative regulator of Th1 cell development and, together with the kinase PDK1, as a positive regulator of Th2 cell development [48]. 

**Figure 3:**
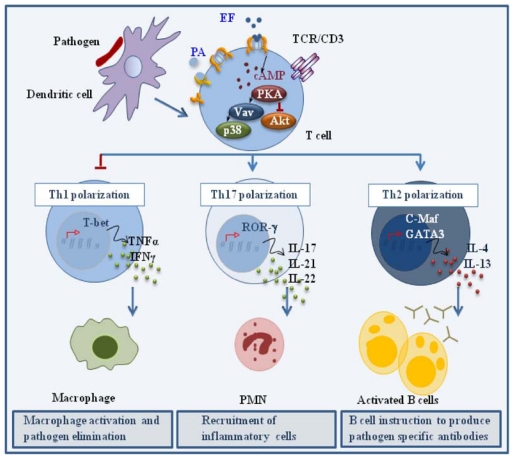
Effects of ET on helper T cell polarization. ET promotes CD4^+ ^T cell polarization to Th2 and Th17 effectors by modulating TCR signaling to promote the expression of the transcription factors c-Maf/GATA-3 and RORγ, which are in turn responsible for the expression of the Th2 driving cytokines IL-4 and IL-13, and the Th17 driving cytokines IL-17, IL-21 and IL-22, respectively.

When co-administered with antigen at low concentrations in the mouse, ET has been shown to act as an adjuvant, enhancing the humoral response by promoting the generation of antigen specific Th2 cells. Although this results, at least in part, from the modulation of cytokine production by APCs [49,50], we have recently reported that ET can directly promote the polarization of human CD4^+^ T cells to Th2 cells in the absence of APC [34]. These experiments were carried out using a concentration of ET that was not immunosuppressive but was able to elicit a modest accumulation of cAMP and activate PKA. When peripheral T cells treated with this low ET concentration were activated by TCR ligation in the absence of any cytokine, they polarized towards the Th2 lineage, as assessed by their cytokine profile and expression of the Th2 trascription factors, c-maf and GATA-3. As expected from the known Th1/Th2 cross-inhibition [39,40,41], Th1 development was impaired. We have recently shown that ET also promotes the robust polarization of CD4^+^ T cells to the Th17 subset, as shown by the pattern of cytokines (IL-23, IL-21, IL-22), cytokine receptors (IL-1R, IL-23R) and transcription factors (RORγ) [51] (Figure 3). Consistent with the Th2 and Th17 polarizing activity of PGE_2_, these effects were not reproduced by a catalytically inactive ET mutant, indicating that they are mediated by the cAMP elevating activity of the toxin. 

Remarkably, early TCR signaling was not impaired in the presence of the immunodeviating concentration of ET, as assessed by CD3ζ phosphorylation and ZAP-70 activation. However the activation of individual signaling mediators implicated in Th1 and Th2 cell development was substantially affected by ET [34]. Specifically, TCR dependent phosphorylation of Akt, a kinase implicated in Th1 cell development and negatively regulated by PKA [48], was found to be impaired in the presence of ET. Conversely, activation of Vav, a guanine nucleotide exchanger responsible for the activation of Rho family GTPases and known to be required for Th2 cell development [52], as well as the downstream stress kinase p38, were found to be enhanced. Hence ET favors Th2 cell development by shaping TCR signaling to selectively modulate the activation of individual specific signaling mediators without an overall modification of the signaling cascade. How ET modulates TCR signaling to promote the polarization of Th17 cells, as well the mechanisms underlying the mixed Th2/Th17 polarizing activity of ET (and PGE_2_), remain to be defined. 

To date the potential immunomodulatory activity of LT on helper T cell polarization has not been addressed. Nevertheless, both the MAP and the stress kinases have been implicated in CD4^+^ T cell lineage commitment, suggesting that LT might also affect this process. 

The Th2 driving activity of ET is likely be relevant to cutaneous anthrax, where there is a limited toxin production and where resolution of infection has been correlated to the development of an antibody response against the toxins. On a more general note, both Th2 and Th17 cells are involved in the development of effective vaccine induced memory responses against infectious diseases [49]. An important implication of the Th2/Th17 immunodeviating activity of ET, which can be reproduced by the unrelated adenylate cyclase toxin of *Bordetella pertussis*, CyaA [34,51], is that cAMP might enhance the efficacy of vaccines by acting on both the humoral and cellular arms of adaptive immunity. In fact, the dual Th2/Th17 polarizing activity of ET could account for the robust humoral and cell-mediated immunity in survivors of anthrax infections [53].

## 6. Conclusions

Although upon infection bacterial pathogens are immediately confronted by the first line defenses provided by the innate immune system, they eventually have to deal with the highly specific and powerful adaptive defenses. Hence the systematic disabling of the innate and adaptive immune defenses appears as an effective survival strategy evolved by *B. anthracis* to achieve successful colonization, particularly in the more common disease presentation, *i.e*., cutaneous anthrax. For this crucial task the bacterium uses two toxins which target signaling mediators that are among the most conserved in eukaryotic cells, from yeast to mammals. The importance of these targets is witnessed by the fact that unrelated pathogens have evolved similar or different mechanisms to ultimately inactivate or subvert their function, as strikingly exemplified by the widespread usage of adenylate cyclases or cAMP modulating toxins as virulence factors [54]. As, such, these toxins provide a unique tool not only to understand the interaction of the pathogen with the host, but to achieve insight into the cellular processes regulated by the toxin targets. For example, cAMP/PKA signaling is crucially implicated in the modulation of TCR signaling, acting in both positive and negative regulatory networks that are subject to a tight spatiotemporal control and which, when subverted, can lead to unwanted oucomes, such as T cell anergy or apoptosis [21]. The underlying mechanisms are finally beginning to be unraveled. As summarized here for *B. anthracis* ET and as reported for *B. pertussis* CyaA [34,51,55], bacterial adenyate cyclase toxins have opened a unique point of observation to elucidate this important biological question. 
